# Effect of exercise training after bariatric surgery: A 5-year follow-up study of a randomized controlled trial

**DOI:** 10.1371/journal.pone.0271561

**Published:** 2022-07-15

**Authors:** Alice Bellicha, Cecile Ciangura, Celina Roda, Adriana Torcivia, Judith Aron-Wisnewsky, Christine Poitou, Jean-Michel Oppert

**Affiliations:** 1 Department of Nutrition, Center for Research on Human Nutrition (CRNH) Ile de France, Pitie-Salpetriere Hospital, AP-HP, Sorbonne University, Paris, France; 2 Nutrition and Obesities: Systemic Approaches (NutriOmics) Team, INSERM, Sorbonne University, Paris, France; 3 Nutritional Epidemiology Research Team (EREN), Inserm U1153, Inrae U1125, Cnam, Epidemiology and Statistics Research Center—University Paris Cité (CRESS), Sorbonne Paris Nord University, Bobigny, France; 4 Health Environmental Risk Assessment (HERA) Team, CRESS, Inserm, INRAE, Université Paris Cité, Paris, France; 5 Faculté de Pharmacie de Paris, Université Paris Cité, Paris, France; 6 Department of Hepato-bilio-pancreatic Digestive Surgery and Liver Transplantation, Pitie-Salpetriere Hospital, AP-HP, Sorbonne University, Paris, France; University of Texas Medical Branch at Galveston, UNITED STATES

## Abstract

**Background and objectives:**

We previously showed in a 6-month randomized controlled trial that resistance training and protein supplementation after bariatric surgery (Roux-en-Y gastric bypass, RYGB) improved muscle strength without significant effect on weight loss and body composition changes. We performed a 5-year follow-up study in these subjects with the aim 1) to assess the long-term effect of this exercise training intervention and 2) to analyze associations between habitual physical activity (PA) and weight regain at 5 years.

**Methods:**

Fifty-four out of 76 initial participants (follow-up rate of 71%) completed the 5-year follow-up examination (controls, n = 17; protein supplementation, n = 22; protein supplementation and resistance training, n = 15). We measured body weight and composition (DXA), lower-limb strength (leg-press one-repetition maximum) and habitual PA (Actigraph accelerometers and self-report). Weight regain at 5 years was considered low when <10% of 12-month weight loss.

**Results:**

Mean (SD) time elapse since RYGB was 5.7 (0.9) y. At 5 years, weight loss was 32.8 (10.1) kg, with a mean weight regain of 5.4 (SD 5.9) kg compared with the 12-month assessment. Moderate-to-vigorous PA (MVPA) assessed by accelerometry did not change significantly compared with pre-surgery values (+5.2 [SD 21.7] min/d, P = 0.059), and only 4 (8.2%) patients reported participation in resistance training. Muscle strength decreased over time (overall mean [SD]: -49.9 [53.5] kg, respectively, P<0.001), with no statistically significant difference between exercise training intervention groups. An interquartile increase in MVPA levels was positively associated with lower weight regain (OR [95% CI]: 3.27 [1.41;9.86]).

**Conclusions:**

Early postoperative participation in a resistance training protocol after bariatric surgery was not associated with improved muscle strength after 5 years of follow-up; however, increasing physical activity of at least moderate intensity may promote weight maintenance after surgery. PA may therefore play an important role in the long-term management of patients with obesity after undergoing bariatric procedure.

## Introduction

The prevalence of severe obesity (defined as a BMI ≥ 40 kg/m^2^) is increasing worldwide, reaching up to 9% of the adult population in the US [1]. Bariatric surgery is recognized as the most effective treatment of severe obesity and is increasingly performed, with no less than 700,000 surgical procedures performed in the world in 2018 [2]. The benefits of bariatric surgery have been extensively described and include substantial and sustained reduction of body weight, improvement in obesity comorbidities, quality of life and physical function, as well as decreased mortality risk [3–6]. However, progressive weight regain is observed as early as 12 to 24 months after surgery, reaching 25% or more of maximal weight loss in half of patients after five years [7, 8]. Weight regain has detrimental effects on obesity comorbidities [9] and health-related quality of life [3] and is therefore a major concern at mid- to long-term after surgery [6].

Exercise training is demonstrated to be an effective strategy for optimizing weight loss and further improving cardiorespiratory and muscular fitness at short term after bariatric surgery [10]. Exercise training is therefore an integral part of recommendations for the follow-up after bariatric surgery [11, 12]. It has been suggested that weight loss and gain in muscle strength may be maintained for several months (i.e. 3 and 12 months) after the end of the post-bariatric exercise training program [10]. This contention was however based on findings from only two studies [13, 14], and needs further confirmation. The effects of detraining have received considerable attention over the last decades in the field of exercise physiology [15, 16]. It is usually accepted that training-induced adaptations (e.g. increased muscle strength, cardiorespiratory fitness or physical function) progressively decline after cessation of exercise training, without however being abolished, especially for muscle strength that usually remains above control values for very long periods [15, 16]. Such findings have been reported in healthy individuals [17, 18] as well as in some clinical populations [17, 19]. However, in the specific context of follow-up after bariatric surgery, the effect of exercise training programs beyond 12 months of follow-up without exercise remains unknown.

The role of habitual physical activity (PA), as assessed by movement sensors (e.g. accelerometers) or self-report (questionnaires), on long-term weight loss after bariatric surgery is not well understood. Several systematic reviews published during the last decade have reported a positive association between habitual PA, or post-operative change in PA, and weight loss in the first years after surgery [20–22]. More recently, a few studies have reported similar associations between PA and weight regain at longer term after surgery (i.e up to 9-year follow-up) [23–26]. However, as pointed out [25], these studies mainly relied on non-validated self-reported PA measures, and assessment of only one type of PA (i.e. moderate-to-vigorous PA [MVPA]) at only one time point. For the design of advanced management strategies, it is of importance to better understand the role of PA in weight maintenance after bariatric surgery. This requires an accurate assessment of a large set of PA and sedentary behavior outcomes, using validated assessment methods in the post-bariatric setting.

We previously published findings of a randomized controlled trial (RCT) combining an exercise training program with protein supplementation in the first 6 months after bariatric surgery which was effective at improving muscle strength, without significantly altering lean body mass loss [27] at 6 months, as compared to the standard of care. The first aim of the present follow-up study was to assess whether this early intervention was able to maintain differences in muscle strength at 5 years after bariatric surgery. A second aim was to investigate whether 5-year accelerometry-assessed and self-report habitual PA and sedentary behavior were associated with weight regain 5 years after bariatric surgery.

## Methods

### Study design and population

We conducted a follow-up study of a single-center, open-label, parallel-group RCT assessing the effect of exercise training on physical fitness and body composition over the first six months after Roux-en-Y Gastric Bypass (RYGB) (clinicaltrials.gov identifier: NCT01113996) [27]. Recruitment took place between May 2010 and December 2014 [27] among patients followed-up at the Department of Nutrition of Pitié-Salpêtrière University Hospital (Assistance Publique-Hôpitaux de Paris) in Paris, France. Patients were contacted by their physician or by the study investigators during planned preoperative hospitalizations or outpatient clinics. Inclusion criteria were female gender, age between 18 and 65 years, living in Paris (France) or its region, and displaying the usual inclusion criteria for bariatric surgery (i.e. body mass index (BMI) ≥ 40 kg/m^2^, or BMI ≥35 kg/m^2^ with at least one obesity comorbidity). The sampling process is described in detail elsewhere [27]. Briefly, from 290 patients assessed for eligibility, 71 patients were not included because they did not meet inclusion criteria and 125 refused to participate. A total of 94 patients were enrolled but 18 were excluded after enrollment because of a surgery path interruption or because sleeve gastrectomy was performed. Therefore, 76 participants were included in the initial trial [27]. Ethical approval was obtained for the initial RCT the Ethics Committee of Pitié-Salpêtrière Hospital (Paris, France), and for the follow-up this cohort from the French Research Ethics Committee of CPP Ile de France-1 N°13533, and from the “Commission nationale de l’informatique et des libertés” No. 1222666. Written informed consent was obtained from all individual participants included in the trial and in the cohort study.

### Randomization and intervention

Patients underwent RYGB and were randomly assigned at the time of surgery to usual care (CON, N = 22), or usual care with oral protein supplementation intake (PRO, N = 31), or usual care, additional protein intake, and supervised resistance training (PRO+EX, N = 23). Interventions have been described in detail elsewhere [27]. Briefly, following both national [28] and international guidelines [11], all patients received usual care in a standardized manner by our multidisciplinary team during planned pre- and post-surgery visits at 1, 3, and 6 months. From the first week post-surgery, protein supplementation was provided to patients included in the PRO and PRO+EX groups in the form of whey-protein-enriched powder (48 g/day of whey protein). From week 6 post-RYGB, patients in the PRO+EX group participated in an exercise training program including 3 sessions per week of resistance training. Patients were initially followed up for six months after RYGB during the RCT [27] and onwards for routine care at 12 months and 5 years in accordance with international recommendations for management of patients after surgery [11, 28]. Five-year follow-up assessment took place between June 2015 and October 2019.

### Measures

#### Body composition assessment

Body weight and body composition were measured before RYGB and 1, 3, 6, 12 months and 5 years after RYGB. Body composition (fat mass, lean body mass) was measured by DXA scanning (Hologic Discovery W, software v12.9; Hologic, Bedford, MA) [27]. Weight loss was expressed as absolute and percent total change in body weight since surgery. Weight change at 5 years was defined as follows: ([body weight at 5 years—body weight at 12 months])/total weight loss at 12 months) x 100. Weight regain at 5 years was considered low when <10% of 12-month weight loss. This threshold of 10% weight regain was chosen because it has previously been found to be associated with the progression of obesity comorbidities [8].

#### Physical fitness assessment

Measures of physical fitness were obtained before RYGB, at 6-month and 5-year follow-up. Isometric maximal grip strength was measured using a handgrip dynamometer (Jamar; Sammons Preston Rolyan, Bolingbrook, Canada) and dynamic lower-limb muscle strength was measured on strength training equipment (leg press), as previously described [27]. The 1-RM (one repetition-maximum) was expressed as absolute values (kilograms) and values relative to body weight and lean body mass. Cardiorespiratory fitness was measured by indirect calorimetry during a graded maximal exercise test on a cycle ergometer [29]. Maximally achieved oxygen consumption (VO_2_peak), determined as the highest attained VO_2_ during the test, was expressed in absolute values and relative to body mass.

#### Habitual PA assessment

Habitual PA and sedentary behavior were measured using the Actigraph GT3x accelerometer (Manufacturing Technology, Inc., FL, USA) before RYGB and at 6-month and 5-year follow-up. The accelerometer was worn at the hip for 7 consecutive days during all waking hours except during water-based activities. Data were considered valid when the accelerometer was worn for at least 4 days during at least 8 h each day [30]. Cutpoints published by Freedson et al. were used to quantify sedentary behavior and light-, moderate-,and vigorous-intensity PA [31]. MVPA bouts were defined as 10 or more consecutive minutes above the MVPA activity count threshold with allowance of 2 min below this threshold [23]. The proportion of MVPA spent in bouts was calculated as: (MVPA spent in bouts [min] / total MVPA [min]) x 100.

At the 5-year follow-up examination, the Sedentary, Transportation and Activity Questionnaire (STAQ) [32] was also used to assess the type and duration of actual leisure-time PA and sedentary behavior, as well as transport-related PA over the past month. This questionnaire was derived from the Recent Physical Activity Questionnaire (RPAQ) [33] with additional specific questions on transport-related activities, and reliability as well as validity were previously reported [32]. Responses were obtained during face-to-face standardized interviews with the same investigator (AB). For both methods, patients were considered as meeting the 2018 US PA guidelines and 2020 WHO Guidelines for aerobic activities when MVPA was ≥ 150 min/week [34, 35].

#### Nutritional status and obesity comorbidities

Blood samples were collected after an overnight fast to measure routine parameters (blood count, blood glucose, protein, prealbumin, albumin, 25-OH vitamin D3, vitamin B12, folate, thiamine) at 6, 12 months and 5 years. Obesity comorbidities were defined through detailed assessment of each patient medical history and medication use, as previously described [27].

#### Outcomes

Based on the protocol of our RCT [27], the primary analysis of this follow-up study was pre- to 5-year post-surgery change in lean body mass, lower-limb muscle strength, cardiorespiratory fitness (VO_2_peak), objectively assessed habitual PA, and nutritional status. Secondary analyses were based on the associations between weight regain at the 5-year follow-up assessment and PA outcomes assessed at the same time point.

#### Statistical analyses

At the time the study was designed, sample sizes were determined a priori based on the detection of a preservation effect in lean body mass loss of one-third in the first six months following bariatric surgery in participants included in the PRO and PRO+EX groups [27]. Setting an overall power to 80% with α at 0.05 yielded an estimate of 75 patients (with 31 in PRO group, 22 in PRO+EX and CON groups) [27].

Categorical data are presented as frequencies and percentages and continuous data as means and standard deviations (SD), or medians and 25th and 75th percentiles (P_25_-P_75_). Linear mixed models were used to estimate and test changes over time. The terms “group,” “time,” and “group × time” were included as fixed effects. Each follow-up wave was added to the model as a dummy variable. The intra-subject correlation between repeated measures was taken into account by including a random intercept at patient‐level. Intention-to-treat (ITT) analyses, involving all randomized patients (N = 54 at 5 years), are presented. Associations between habitual PA outcomes assessed at 5 years with accelerometer or self-report measures and weight regain at 5 years follow-up (reference: ≥ 10%, and <10%) were analyzed using logistic regression models. Accelerometer wear time at 5 years and the corresponding baseline preoperative value were included as covariables in the models when analyzing accelerometry data. Odds-ratios (OR) and their 95% confidence intervals (95% CI) representing the association of an interquartile range (IQR) increase in PA levels with weight regain are estimated. Two-sided P-values are reported. Results were considered significant at P < 0.05. Statistical analyses were performed with Stata software (release 13; Stata Corp., College Station, Texas).

## Results

### Characteristics of participants, body composition, physical fitness and activity before and 5 years after RYGB in the whole group

**Fig 1:**
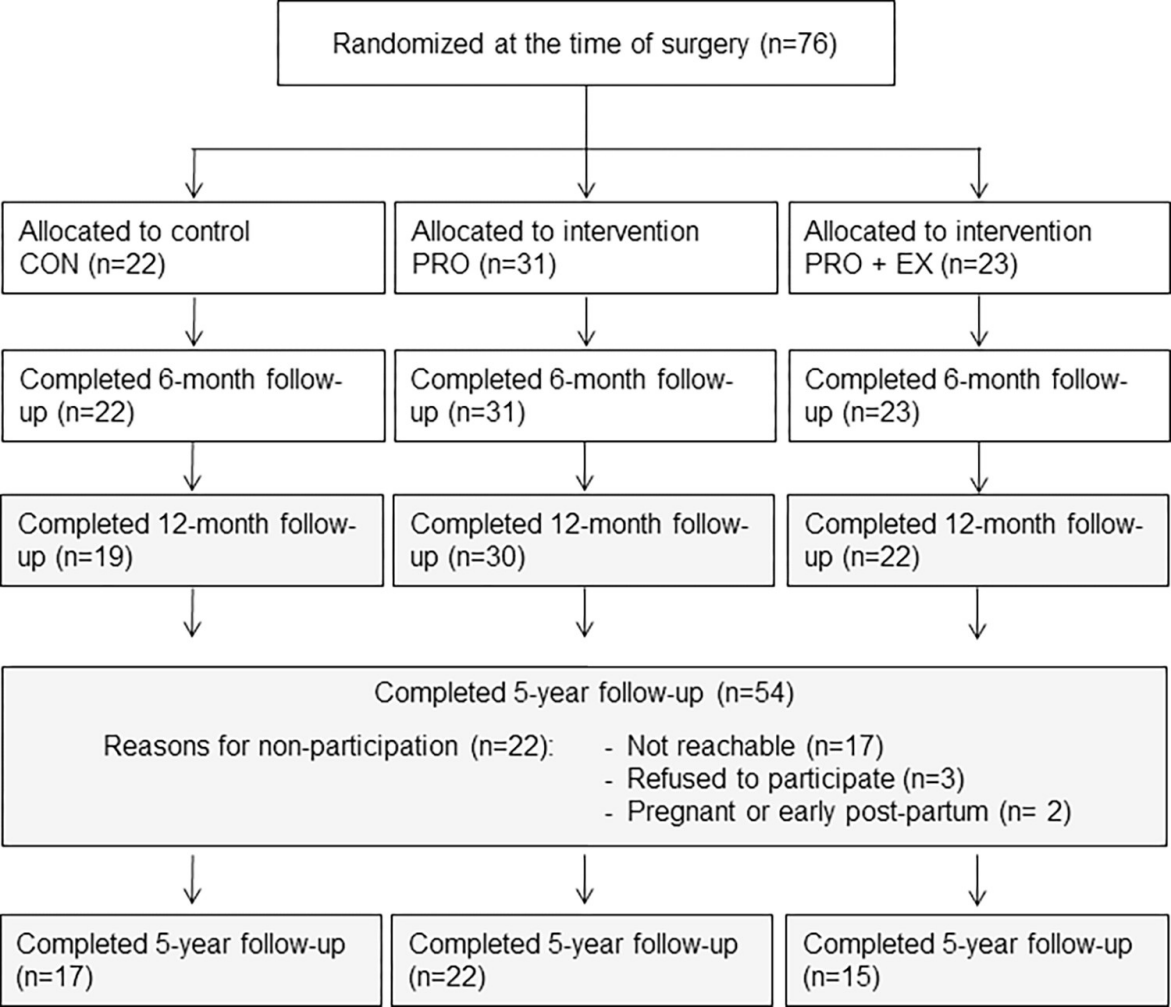
Flow chart of the follow-up study.

Out of 76 patients included in the trial, data from 54 patients (follow-up rate of 71%) were available at 5 years after RYGB (Fig 1). Characteristics of these 54 patients before and 5 years after RYGB are presented in Table 1. Time elapse between the time of bariatric surgery and the time of the last follow-up was 5.7 (SD 0.9) y (min-max: 4.0–8.4 y), with no significant difference between intervention groups. No significant difference was observed in terms of preoperative data between the 54 patients included in the 5-year follow-up assessment and the 22 patients lost to follow-up (S1 Table).

**Table 1:** Characteristics of patients before and 5 years after RYGB (whole sample with intervention groups combined, N = 54).

	N	Before RYGB	N	5 years after RYGB
Age, years	54	43.5 (9.9)	54	49.4 (9.8)
**Anthropometry and body composition**				
Body weight, kg	54	117.2 (15.7)	54	84.4 (14.8)
BMI, kg/m^2^	54	44.2 (5.4)	54	31.9 (5.6)
Body fat, %	53	49.9 (3.8)	53	42.8 (5.4)
LBM, kg	53	55.8 (6.7)	53	46.2 (5.7)
**Comorbidities**	54		54	
Type 2 diabetes, N (%)		17 (31.5%)		9 (16.7%)
Sleep apnea syndrome, N (%)		31 (57.4%)		5 (9.3%)
Hypertension, N (%)		18 (33.3%)		10 (18.5%)
**Physical fitness**				
Handgrip strength, kgF	54	31.1 (7.0)	43	31.5 (4.9)
Leg-press 1-RM				
Absolute, kg	53	182.6 (51.7)	40	129.1 (40.7)
Relative to body weight, kg/kg	53	1.57 (0.47)		1.53 (0.36)
VO_2_peak				
Absolute, L/min	51	2.1 (0.5)	37	1.64 (0.59)
Relative to body weight, mL/min/kg	51	18.2 (4.3)	37	19.5 (6.5)
**Accelerometry-assessed PA**	50		48	
Accelerometer wear time, min/d		798 (737;847)		804 (762; 877)
Counts per minute		292 (229;368)		308 (253;378)
Sedentary time, min/d		486 (434;538)		498 (439;554)
Total PA, min/d		303 (256;378)		302 (252;383)
Light-intensity PA, min/d		267 (228;344)		265 (226;360)
Moderate-intensity PA, min/d		24.7 (15.6;35.9)		29.1 (19.3;39.3)
Vigorous-intensity PA, min/d		0.0 (0.0;0.0)		0.0 (0.0;0.1)
MVPA, min/d		24.7 (15.6;36.0)		29.1 (19.5;40.0)
MVPA spent in bouts, min/week		25.0 (0.0;84.8)		47.5 (0.0;133.0)
MVPA bouts, n/d		0.3 (0.0;0.8)		0.4 (0.0;6.1)
Proportion of total MVPA spent in bouts, %		10.9 (0.0;26.4)		12.4 (0.0;30.8)
Meet PA guidelines, N (%)		30 (60.0%)		33 (68.8%)
**Self-reported PA**	NA		49	
Leisure-time sedentary behavior, min/d		NA		234 (168;330)
TV time, min/d		NA		126 (90;150)
Leisure-time moderate-intensity PA, min/d		NA		13.9 (0.0;32.7)
Leisure-time vigorous-intensity PA, min/d		NA		0.0 (0.0;3.0)
Leisure-time MVPA, min/d		NA		16.6 (0.0;46.1)
Transport-related PA (walking and cycling)		NA		14.3 (2.1;34.3)
Total MVPA, min/d		NA		40.9 (18.8;66.4)
Meet PA guidelines, N (%)		NA		35 (71.4%)

Data are frequencies (percentages) or means (standard deviations) or medians (25^th^ and 75^th^ percentiles).

Abbreviations. 1-RM, one repetition maximum; BMI, body mass index; LBM, lean body mass; MVPA, moderate-to-vigorous physical activity; TV, television.

### Changes in body composition, physical fitness and activity, and nutritional status 5 years after RYGB by intervention group

Table 2 shows changes in anthropometry, body composition, physical fitness, habitual PA and nutritional status 5 years after RYGB according to treatment groups in ITT analysis. A significant time effect was observed for most outcomes but no group x time interaction was found. Overall, compared to pre-surgery values, 5-year post-RYGB body weight was decreased by 32.8 (SD 10.1) kg, in parallel with a decrease in fat mass (-22.3 [SD 8.0 kg]) and in lean body mass (-9.6 [SD 3.8] kg). Muscle strength expressed as absolute and relative values decreased over time by 49.9 (SD 53.5) kg and -0.02 (SD 0.49) kg/kg, respectively. Absolute values of VO_2_max decreased over time (-0.43 [SD 0.54] L/min) whereas relative values increased (+1.6 [SD 6.6] mL/kg/min). Total MVPA measured by accelerometry tended to increase by 5.2 (SD 21.7) min/d (P = 0.059).

**Table 2:** Changes in body composition, physical fitness, habitual physical activity and nutritional status 5 years after RYGB.

		Change from baseline (95% CI)			P-value		
	**N**	**CON N = 17**	**PRO N = 22**	**PRO + EX N = 15**	Group	Time	Group by time
**Anthropometry and body composition**							
Body weight, kg	54	-32.1 (-35.5;-28.8)	-32.4 (-35.3;-29.4)	-34.0 (-37.6;-30.5)	0.503	**<0.001**	0.991
BMI, kg/m^2^	54	-12.1 (-13.3;-10.9)	-12.0 (-13.1;-11.0)	-13.0 (-14.3;-11.7)	0.233	**<0.001**	0.969
Fat mass, kg	54	-22.1 (-24.8;-19.3)	-21.0 (-23.5;-18.5)	-23.8 (-26.8;-20.8)	0.160	**<0.001**	0.854
Lean body mass, kg	54	-10.0 (-11.4;-8.6)	-9.8 (-11.0;-8.5)	-9.1 (-10.6;-7.5)	0.987	**<0.001**	0.964
**Muscle strength**							
Handgrip, kgF	54	0.9 (-1.8;3.5)	-1.0 (-3.3;1.4)	3.1 (-0.2;6.3)	0.127	**<0.001**	0.342
Leg-press 1-RM							
Absolute, kg	51	-49.9 (-81.0;-18.8)	-44.4 (-71.5;-17;3)	-55.5 (-90.1;-20.9)	**0.047**	**<0.001**	0.806
Relative to body weight, kg/kg	51	-0.1 (-0.4;0.2)	0.1 (-0.2;0.3)	0.1 (-0.3;0.3)	0.192	**<0.001**	0.589
Relative to lower limb LBM, kg/kgLBM	51	-1.4 (-3.1;0.4)	-0.7 (-2.2;0.9)	-0.9 (-2.9;1.0)	0.348	**0.008**	0.618
**Cardiorespiratory fitness**							
VO_2_ peak							
Absolute, L/min	53	-0.4 (-0.7;-0.2)	-0.4 (-0.7; -0.2)	-0.4 (-0.7;-0.1)	0.425	**<0.001**	0.627
Relative to body weight, mL/min/kg	53	0.8 (-2.3;4.0)	2.5 (-0.4;5.3)	1.5 (-1.9;4.9)	0.203	**0.014**	0.784
Relative to LBM, mL/min/kgLBM	53	-2.8 (-8.6;3.0)	-1.0 (-6.2;4.3)	-2.1 (-8.5;4.2)	0.432	0.364	0.737
**Habitual physical activity**							
Wear time, min/d	52	16.3 (-30.5;63.1)	38.0 (-3.4;79.4)	48.2 (-4.5;100.9)	0.596	**0.010**	0.629
Sedentary time, min/d	52	20.5 (-24.0;65.0)	34.2 (-5.2;73.7)	17.6 (-32.5;67.6)	**0.049**	0.058	0.853
Counts per minute	52	-8.3 (-69.0;52.5)	15.3 (-38.8;69.3)	54.2 (-14.2;122.6)	0.145	0.270	0.483
Light-intensity PA, min/d	52	-5.1 (-41.7;31.5)	-0.4 (-32.9;32.1)	18.3 (-22.9;59.5)	0.325	0.614	0.303
MVPA, min/d	52	0.1 (-10.0;10.1)	3.1 (-5.8;12.1)	12.4 (1.1;23.8)	0.303	0.059	0.353
MVPA in ≥10-min bouts, min/wk	52	13.8 (-45.7;73.4)	23.7 (-29.3;76.7)	44.7 (-22.3;111.7)	0.263	0.160	0.745
**Nutritional status**							
Hemoglobin, g/dL	54	-0.2 (-0.7;0.2)	-0.2 (-0.6; 0.2)	-0.4 (-0.9;0.1)	0.346	**0.043**	0.975
Proteinemia, g/L	54	-4.4 (-6.4;-2.4)	-2.8 (-4.6;-1.0)	-2.2 (-4.4;-0.4)	0.483	**<0.001**	0.726
Albumin, g/L	54	4.6 (3.0;6.3)	5.4 (3.9;6.8)	5.3 (3.6;7.1)	0.367	**<0.001**	0.967
Prealbumin, g/L	54	-0.02 (-0.04;-0.01)	-0.01 (-0.02;0.01)	-0.02 (-0.04;-0.01)	0.637	**<0.001**	0.781
25(OH) Vitamin D3, ng/mL	54	11.1 (5.4;16.8)	8.9 (3.9;14.0)	11.9 (5.9;17.8)	0.855	**<0.001**	0.970
Thiamine, nmol/L	54	50.7 (17.9;83.4)	22.6 (-4.9;50.1)	35.5 (2.3;68.6)	0.600	**<0.001**	0.339
Erythrocyte folate, nmol/L	54	512.1 (280.4;743.8)	676.6 (474,5;878.6)	924.7 (664.1;1185.3)	0.209	**<0.001**	0.244
B12, pmol/L	54	-173.3 (-238.9;-107.7)	-123.5 (-182.9;-64.2)	-129.5 (-200.9;-58.1)	0.856	**<0.001**	0.820

P-values for group, time, and interaction (group × time) terms in mixed models; bold values indicate significance with P < 0.05.

1-RM, one-repetition maximum; CON, control group; PRO, protein intake group; PRO+EX, protein intake and supervised strength training group; LBM, lean body mass; MVPA, moderate-to-vigorous physical activity; PA, physical activity.

### Body weight and body composition trajectories over 5 years after RYGB

S1 Fig illustrates changes in body weight, fat mass and lean body mass in the whole sample (groups combined) from 1 month to 5 years after RYGB. At 12 months post-RYGB, body weight decreased by 37.9 (SD 9.3) kg (P<0.001) compared to preoperative values. Between 12 months and 5 years post-RYGB, body weight increased by 5.4 (SD 5.9) kg (P<0.001), which corresponds to a mean weight regain of 14.9 (SD 17.7) % of the total amount of weight lost at 12 months. Fat mass decreased by 27.4 (SD 7.8) kg at 12 months and increased by 5.5 (SD 5.4) kg between the 12-month and 5-year follow-up (all P<0.001). Lean mass loss decreased by 9.7 (SD 3.3) kg at 12 months (P<0.001) and did not change significantly thereafter (+0.2 [SD 2.6] kg). No significant difference was observed between groups in the change of body weight and body composition over time from 1 month to 5 years (S2 Table).

### Associations between weight regain and change in habitual PA after RYGB

**Fig 2:**
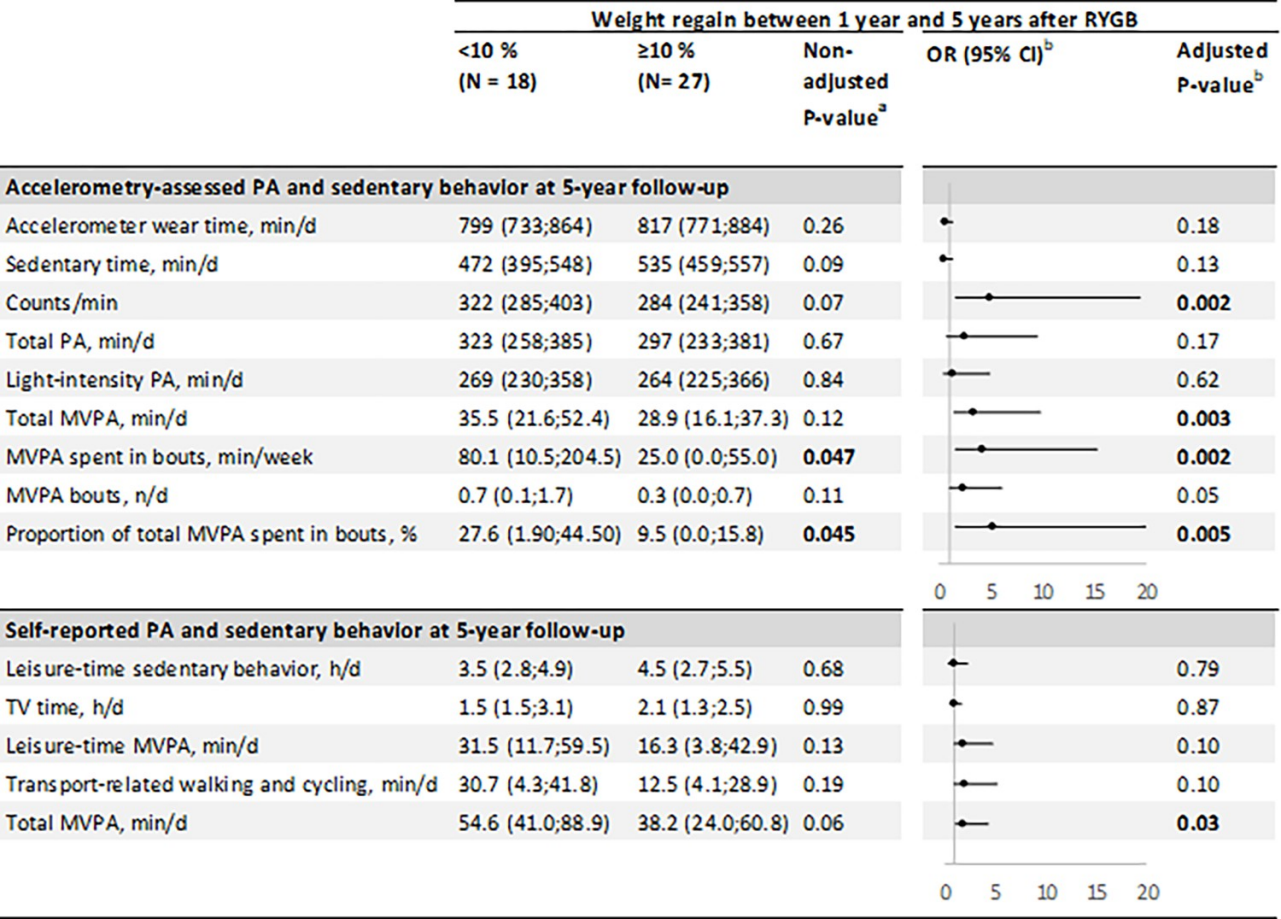
Associations between weight regain 5 years after RYGB and changes in PA outcomes. Data are medians (25^th^ and 75^th^ percentiles). ^a^ Non-adjusted P-value from Wilcoxon rank sum test. ^b^ Logistic regression with accelerometer wear time and baseline preoperative data as covariables (only for accelerometry data). Odds-ratios represent the association between an interquartile range (IQR) increase in PA levels and weight regain (<10% vs. ≥10%). Error bars represent the 95% confidence interval of the OR. Bold values indicate significance with P < 0.05. Self-reported total MVPA is calculated as the sum of leisure-time MVPA and transport-related walking and cycling. Abbreviations: MVPA, moderate-to-vigorous PA.

Weight regain at the 5-year follow-up time point after RYGB was ≥ 10% in 32 (61.5%) patients and <10% in 20 (38.5%) patients. Total percent weight loss since surgery significantly differed between these two groups (-24.2 [SD 7.6] % vs. -33.3% [SD 4.6] %, respectively, P<0.001). However, no significant difference was observed between the two weight regain groups in terms of preoperative characteristics (body weight, percent body fat, frequency of obesity comorbidities), intervention group, RYGB-induced weight loss and changes in body composition at 1-year follow-up, or time elapse between surgery and last follow-up (S3 Table). Valid accelerometry data were obtained before and 5 years after RYGB in 45 patients, of whom 27 (60%) experienced a ≥10% weight regain. Fig 2 presents the associations between the weight regain and PA outcomes. Positive associations were found between a lower weight regain (< 10%) and accelerometry-assessed counts/min (OR [95% CI]: 4.90 [1.62;19.5] per IQR increase of 122.8 counts/min), total MVPA (OR [95% CI]: 3.27 [1.41;9.86] per IQR increase of 20.9 min/d), MVPA spent in bouts (OR [95% CI]: 4.13 [1.54;15.22] per IQR increase of 108.5 min/d) and proportion of total MVPA spent in bouts (OR [95% CI]: 5.17 [1.60;21.05] per IQR increase of 31.8%). The same association was found with self-reported total MVPA (OR [95% CI]: 1.90 [1.04;4.36] per IQR increase of 42.8 min/d).

### Habitual PA 5 years after RYGB

**Fig 3:**
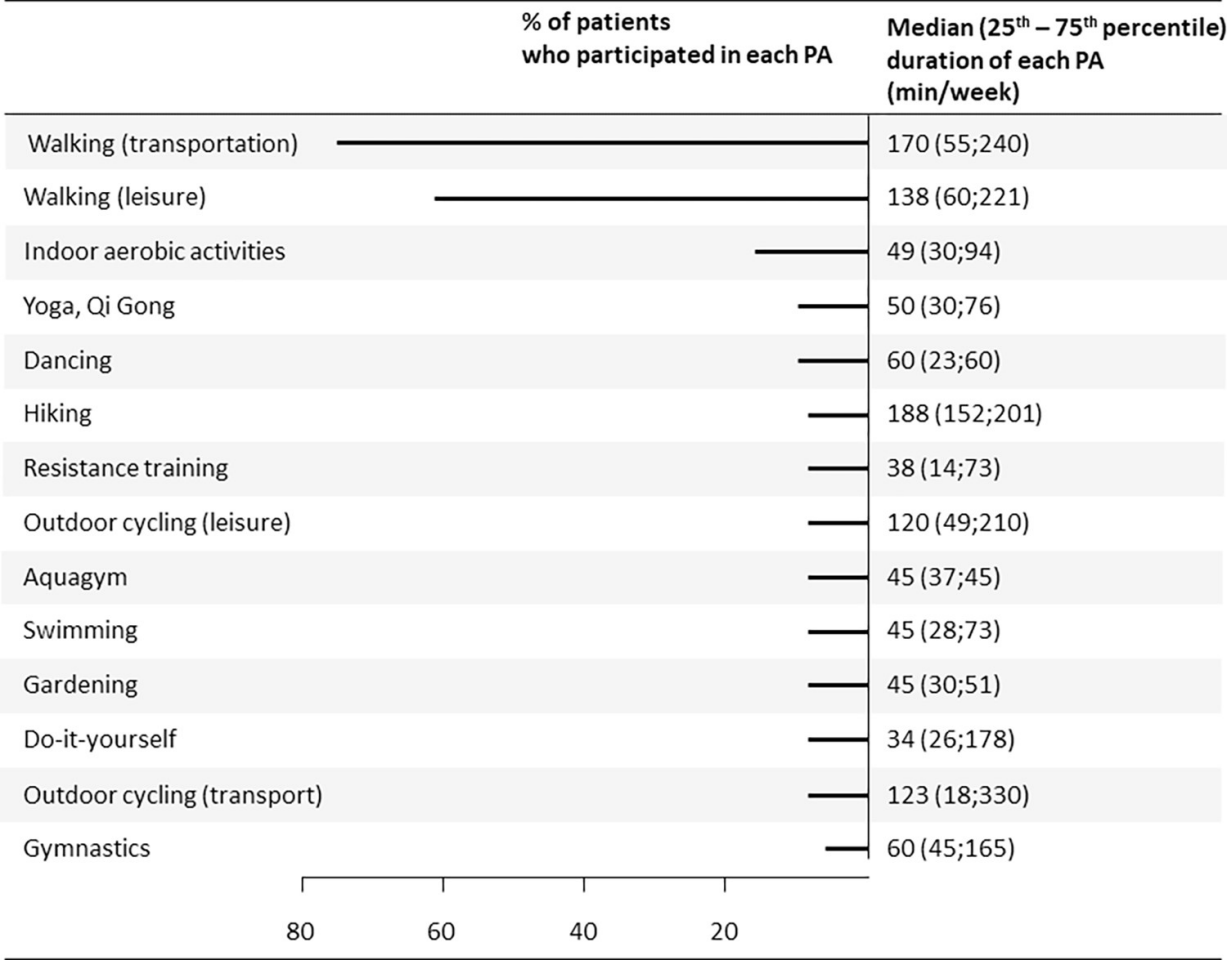
Type and duration of leisure-time activities performed 5 years after RYGB. Jogging, indoor aerobic exercise, climbing were reported by one or two patients and are therefore not presented in this figure.

When analyzing accelerometry and self-reported data, 69% and 71% of participants were found to meet aerobic PA guidelines at 5 years after RYGB, respectively (Table 1). Walking for transportation and for leisure were the two most frequently reported PA, with 37 (75.5%) and 30 (61.1%) patients reporting participation in these activities over the past month, respectively (Fig 3). Only 4 (8.2%) patients reported participation in resistance training.

## Discussion

In this study, we provide data on body composition, physical fitness and activity, and nutritional status of patients examined 5 years after bariatric surgery, those patients having been included initially in an RCT with exercise training and protein supplementation in the first 6 months after surgery. An important previous result from that RCT was that muscle strength was found increased with supervised resistance training and protein supplementation compared to usual care or usual with protein supplementation but no exercise [27]. At 5 years however, the present study shows no significant difference in muscle strength between groups. This is in agreement with previous studies that reported a progressive decline in training-induced adaptations after training cessation [16]. This is however in contrast with data from our recent meta-analysis [10] which suggested that exercise-induced gain in muscle strength may be sustained after bariatric surgery up to 3 and 12 months of follow-up without exercise [13, 14]. Collectively, these findings suggest that increased muscle strength may be maintained for several months after an exercise training program in patients who underwent bariatric surgery, but would return to the baseline level at longer term.

Independent of intervention groups, there was in the whole sample a small but significant decrease in relative values of muscle strength 5 years after bariatric surgery compared to preoperative values. Previous studies have reported an increase in relative strength occurring in parallel with weight loss during the first 12 months after surgery [27, 36, 37], which suggests that the initial gain in relative strength following bariatric surgery is difficult to maintain over the longer term in middle-aged women. Muscle strength expressed relative to body weight is considered an interesting way to estimate an individual’s functional performance [38]. Therefore, the decrease in relative strength observed 5 years after RYGB despite sustained weight loss could be associated with a decline in physical function, which may become a concern at long term after bariatric surgery, especially in patients who are getting older. Such a decrease in muscle strength can be primarily attributed to the loss of lean body mass, which itself is due to the dramatic decrease in protein intake observed in the first months following bariatric surgery [27, 39]. Interestingly, our study and others [40, 41] showed that rates of lean body mass loss were higher during the first 3 to 6 months after RYGB, characterized by very low protein intake [27, 39], followed by a more gradual decrease up to 12 months. Our initial trial showed that combining resistance training and protein supplementation was however insufficient to counteract the effect of bariatric surgery on lean body mass loss during the first post-operative months [27]. Beyond insufficient protein intake, weight loss itself may play a role in the loss of lean body mass. Indeed, it has been hypothesized that additional body weight may provide a muscle overload stimulus during physical activity, especially when using anti-gravitational muscles, resulting in increased muscle strength in people with obesity [42, 43]. It is therefore likely that the surgery-induced weight loss, by reducing such overload stimulus, will result in a progressive loss of muscle strength after bariatric surgery.

The most effective mean to improve muscular fitness is through resistance training, whether performed alone or combined with aerobic training [44]. However, even though resistance training is recommended in the follow-up after bariatric surgery [11, 12], less than 10% of the patients included in our study reported participation in resistance training at the 5-year follow-up assessment. Insufficient training stimulus, resulting from weight loss and from low participation in resistance training, may therefore be the main explanation for the large loss of muscle strength over the 5-year follow-up. Although we did not assess the participation in resistance training during the follow-up, our results suggest that participation to a structured resistance training program did not translate into long-term adherence to resistance training recommendations. According to a recent systematic review, the main barriers to physical activity in people with obesity are lack of motivation, pain or physical discomfort, lack of time, lack of social support and lack of access to equipment, facilities or professionals [45]. Besides, resistance training appears to be less often identified as a preferred type of physical activity in people with obesity, as compared to walking, cycling, or water-based activities [45]. This may explain why a lower proportion of patients reported participation in resistance training (8%), compared to those meeting the aerobic PA guidelines of 150 min per week of MVPA (60%). Our findings highlight the need to include supervised resistance training programs in routine care after bariatric surgery to optimize its long-term benefits on muscular fitness.

The second aim of this study was to analyze the associations between habitual PA and weight regain at 5 years after RYGB. Weight regain is major concern after bariatric surgery because it can lead to the recurrence of obesity comorbidities, in particular type 2 diabetes [9, 46], decreased quality of life [3], and increasing demand for revisional surgery [47, 48]. We found significant associations between MVPA and low weight regain (i.e. <10% of weight lost at 12 months), whether MVPA was assessed by accelerometry or by self-report. In contrast, sedentary time and light-intensity PA were not significantly associated with weight regain. The strongest association between weight regain and PA was found for the proportion of MVPA spent in ≥ 10 min bouts, which was almost three times larger in the group of patients who experienced a < 10% weight regain. Overall, these findings add to the body of evidence suggesting that PA may help prevent weight regain after bariatric surgery and that increasing both duration and intensity of PA bouts are of importance. This would be in line with the known effects of PA for weight maintenance after dietary-induced weight loss [49]. Secondary analyses of RCTs have indeed consistently showed that individuals who engaged in greater amounts of exercise during the weight maintenance phase experienced less weight regain, with a dose–response relationship, and that large volume of PA (≥250 min/week) may be needed [50–52]. Our results suggest that a similar effect may be expected in the context of bariatric surgery. Further studies are however needed to identify the optimal duration of MVPA to help with long-term weight maintenance in this specific context.

Although 60% of the patients included in our study were found to meet the aerobic PA guidelines of 150 min per week of MVPA at 5 years after surgery, only a quarter of them were able to reach the amount of 250 min per week of MVPA that is recommended to prevent weight regain after dietary-induced weight loss [12]. Efforts are therefore needed to better promote PA among post-bariatric patients. In this setting, exercise training programs have proven effective at improving body composition and physical fitness but they do not seem to result in increased PA in daily life [10]. Access to supervised exercise training programs and behavioral interventions may therefore be needed to increase daily PA, promote weight maintenance and optimize the health benefits of bariatric surgery.

Our findings showed that accelerometers and interview-based questionnaires are useful tools to identify inactive patients who are at greater risk of weight regain after bariatric surgery. Accelerometers provide a more accurate measure of the time spent in different intensities of PA [53], which may explain why weight regain was more strongly associated with objectively-measured PA. In the post-bariatric context, the use of self-administered questionnaires has been found to greatly overestimate PA [30, 54], which encouraged us to use interview-based questionnaires. Self-report methods are complementary to accelerometers in that they provide information on the type and context of PA that are essential for tailoring physical activity advice. Both methods should therefore be used in combination for a thorough assessment of PA, which will be useful for patient management and for research purposes. Their use in clinical settings is however limited by time and resources constraints [55] but we can expect that new technologies such as smartphones, smartwatches and activity trackers, all of which contain native accelerometers at a lower cost than research-grade accelerometers, will facilitate routine assessment of PA in such clinical settings.

Strengths of our study include the homogeneous sample of patients receiving RYGB who were included in our previous RCT, the monitoring of patients up to 5 years after surgery, and the assessment of body composition, physical fitness and PA outcomes using validated methods. Some limitations should however be mentioned. First, the generalizability of our findings may be limited by the small sample size, the strict inclusion criteria (i.e. only women receiving RYGB) and the loss of patients to follow-up. Although the follow-up rate of 71% at 5 years is relatively high and consistent with previous exercise interventions conducted in similar settings with a 24-month follow-up [14], it is likely that post-surgery outcomes may have differed in patients lost to follow-up [56]. Second, we did not assess eating behavior, which plays an important role in weight regain after bariatric surgery [57]. Given the benefits of PA on eating behavior in general [58], we can hypothesize that PA modulates the relation between eating behavior and weight regain after bariatric surgery. Although a previous study reported no significant association between self-reported PA and eating behavior after bariatric surgery [59], further investigation on this issue will be of interest. Finally, we measured body weight 12 months after RYGB but were not able to determine the exact time when the nadir weight occurred. However, data from the SOS Study showed that maximal weight loss was obtained on average one year after RYGB [7], and previous analyses from our group showed the relevance of assessing 12-month body weight when investigating 5-year weight regain and relapse of metabolic comorbidities [9].

## Conclusions

The initial favorable effect of exercise training and protein supplementation in increasing muscle strength after bariatric surgery was not sustained after a 5-year follow-up. Overall, muscle strength decreased from pre-surgery to 5-year post-surgery, with potential negative consequences on physical function, especially in patients who are getting older. Independent of intervention groups during the first 6 postoperative months, habitual MVPA assessed by accelerometry and self-report 5 years after bariatric surgery was associated with lower weight regain and may therefore enhance the medium- to long-term benefits of bariatric surgery. However, at 5 years post-surgery, 61% and 10% of participants were found to meet aerobic PA guidelines and to participate in resistance training, respectively, suggesting the importance of ongoing promotion of both aerobic PA and resistance training in patients who undergo bariatric surgery.

## Supporting information

S1 Table Comparisons of baseline preoperative characteristics between participants completing the 5-year assessment (N = 54) and participants lost to follow-up (N = 22). Data are frequencies (percentages) or means (standard deviations) or medians (25th and 75th percentiles). P-value from Wilcoxon rank sum test, χ2 test or Fisher exact test. Abbreviations. 1-RM, one repetition maximum; BMI, body mass index; LBM, lean body mass; MVPA, moderate-to-vigorous physical activity.
(DOCX)

S2 Table Changes in body weight, fat mass and lean body mass after RYGB according to intervention groups (N = 54). Data are means (95% CI). Abbreviations: CON, control group; PRO, protein intake group; PRO + EX, protein intake and supervised strength training group. No significant difference was observed between groups at each time point.
(DOCX)

S3 Table Comparisons between categories of weight regain between 1 year and 5 years after RYGB in terms of preoperative characteristics or RYGB-induced changes in body weight and body composition. Non-adjusted P-value from Wilcoxon rank sum test, χ^2^ test or Fisher exact test.
(DOCX)

S1 Fig Changes in body weight, fat mass and lean body mass over 5 years after RYGB independent of intervention groups. P values for time effect in mixed models. ^a^ Significantly different from preoperative value. ^b^ Significantly different from 1-month follow-up post-surgery. ^c^ Significantly different from 3-month follow-up post-surgery. ^d^ Significantly different from 6-month follow-up post-surgery. ^e^ Significantly different from 12-month follow-up post-surgery.
(TIF)
