# Supplementary material for: Effect of exercise training after bariatric surgery: A 5-year follow-up study of a randomized controlled trial
Source: PLoS One. 2022 Jul 15;17(7):e0271561. doi: 10.1371/journal.pone.0271561 (PMC9286216; doi:10.1371/journal.pone.0271561)
Supplement: S1 Table — Data are frequencies (percentages) or means (standard deviations) or medians (25th and 75th percentiles). P-value from Wilcoxon rank sum test, χ2 test or Fisher exact test. Abbreviations. 1-RM, one repetition maximum; BMI, body mass index; LBM, lean body mass; MVPA, moderate-to-vigorous physical activity. (DOCX) [file pone.0271561.s001.docx]

**S1 Table. Comparisons of baseline preoperative characteristics between participants completing the 5-year assessment (N= 54) and participants lost to follow-up (N= 22)**

|  | **Patients completing**  **5-year follow-up (N= 54)** | **Patients lost to follow-up (N= 22)** | **P-value** |
| --- | --- | --- | --- |
| Age, years | 43.5 (9.9) | 39.7 (9.6) | 0.16 |
| **Anthropometry and body composition** |  |  |  |
| Body weight, kg | 117.2 (15.7) | 113.9 (17.6) | 0.40 |
| BMI, kg/m^2^ | 44.2 (5.4) | 43.3 (6.6) | 0.28 |
| Body fat, % | 49.9 (3.8) | 50.4 (4.5) | 0.56 |
| LBM, kg | 55.8 (6.7) | 54.9 (6.8) | 0.71 |
| **Comorbidities** |  |  |  |
| Type 2 diabetes, N (%) | 17 (31.5%) | 4 (18.2%) | 0.37 |
| Sleep apnea syndrome, N (%) | 31 (57.4%) | 8 (36.4%) | 0.16 |
| Hypertension, N (%) | 18 (33.3%) | 4 (18.2%) | 0.30 |
| **Physical fitness** |  |  |  |
| Handgrip strength, kgF | 31.1 (7.0) | 32.1 (4.7) | 0.40 |
| Lower limb 1-RM |  |  |  |
| Absolute, kg | 182.6 (51.7) | 183.2 (47.8) | 0.77 |
| Relative to body weight, kg/kg | 1.57 (0.47) | 1.65 (0.49) | 0.40 |
| VO_2_peak |  |  |  |
| Absolute, L/min | 2.1 (0.5) | 2.1 (0.5) | 0.71 |
| Relative to body weight, mL/min/kg | 18.2 (4.3) | 18.3 (3.9) | 0.52 |
| **Accelerometry-assessed PA** |  |  |  |
| Accelerometer wear time, min/d | 798 (737;847) | 771 (755;819) | 0.56 |
| Counts per minute | 292 (229;368) | 312 (284;350) | 0.34 |
| Sedentary time, min/d | 486 (434;538) | 452 (397;506) | 0.09 |
| Total PA, min/d | 303 (256;378) | 343 (291;363) | 0.18 |
| Light-intensity PA, min/d | 267 (228;344) | 318 (263;345) | 0.15 |
| MVPA, min/d | 24.7 (15.6;36.0) | 24.0 (17.0;34.1) | 0.88 |
| MVPA spent in bouts, min/week | 25.0 (0.0;84.8) | 20.0 (0.0;65.4) | 0.61 |
| MVPA bouts, n/d | 0.3 (0.0;0.8) | 0.2 (0.0;0.6) | 0.54 |
| Proportion of total MVPA spent in bouts, % | 10.9 (0.0;26.4) | 7.7 (0.0;0.17) | 0.43 |
| Meet PA guidelines, N (%) | 30 (60.0%) | 11 (55%) | 0.91 |

Data are frequencies (percentages) or means (standard deviations) or medians (25^th^ and 75^th^ percentiles). P-value from Wilcoxon rank sum test, χ^2^ test or Fisher exact test.

Abbreviations. 1-RM, one repetition maximum; BMI, body mass index; LBM, lean body mass; MVPA, moderate-to-vigorous physical activity.
