# Supplementary material for: Effect of exercise training after bariatric surgery: A 5-year follow-up study of a randomized controlled trial
Source: PLoS One. 2022 Jul 15;17(7):e0271561. doi: 10.1371/journal.pone.0271561 (PMC9286216; doi:10.1371/journal.pone.0271561)
Supplement: S2 Table — Data are means (95% CI). Abbreviations: CON, control group; PRO, protein intake group; PRO + EX, protein intake and supervised strength training group. No significant difference was observed between groups at each time point. (DOCX) [file pone.0271561.s002.docx]

**S2 Table. Changes in body weight, fat mass and lean body mass after RYGB according to intervention groups (N=54)**

|  | **CON**  **N = 17** | **PRO**  **N=22** | **PRO + EX**  **N=15** |
| --- | --- | --- | --- |
| **Body weight, kg** |  |  |  |
| 1 month | -10.1 (-13.4;-6.7) | -9.6 (-12.5;-6.7) | -10.6 (-14.1;-7.0) |
| 3 months | -18.1 (-21.4;-14.7) | -19.3 (-22.2;-16.3) | -17.9 (-21.5;-14.3) |
| 6 months | -27.7 (-31.1;-24.4) | -28.7 (-31.6;-25.8) | -28.6 (-32.2;-25.1) |
| 12 months | -38.0 (-41.4;-34.6) | -37.7 (-40.6;-34.8) | -38.1 (-41.7;-34.5) |
| 5 years | -32.1 (-35.5;-28.8) | -32.4 (-35.3;-29.4) | -34.0 (-37.6;-30.5) |
| **Fat mass, kg** |  |  |  |
| 1 month | -5.2 (-8.0;-2.4) | -3.7 (-6.1;-1.2) | -4.4 (-7.4;-1.4) |
| 3 months | -11.6 (-14.4;-8.8) | -12.3 (-14.8;-9.8) | -11.4 (-14.3;-8.4) |
| 6 months | -19.8 (-22.6;-17.0) | -19.9 (-22.4;-17.5) | -20.0 (-22.9;-17.0) |
| 12 months | -28.1 (-31.0;-25.3) | -26.8 (-29.2;-24.3) | -27.4 (-30.4;-24.3) |
| 5 years | -22.1 (-24.8;-19.3) | -21.0 (-23.5;-18.5) | -23.8 (-26.8;-20.8) |
| **Lean body mass, kg** |  |  |  |
| 1 month | -5.5 (-6.9;-4.1) | -5.7 (-7.0;-4.5) | -5.8 (-7.3;-4.4) |
| 3 months | -7.1 (-8.5;-5.7) | -6.7 (-8.0;-5.5) | -6.0 (-7.5;-4.5) |
| 6 months | -8.6 (-10.0;-7.2) | -8.4 (-9.7;-7.2) | -8.2 (-9.7;-6.7) |
| 12 months | -9.5 (-10.9;-8.1) | -9.9 (-11.2;-8.7) | -9.5 (-11.0;-8.0) |
| 5 years | -10.0 (-11.4;-8.6) | -9.8 (-11.0;-8.5) | -9.1 (-10.6;-7.6) |

Data are means (95% CI).

Abbreviations: CON, control group; PRO, protein intake group; PRO + EX, protein intake and supervised strength training group.

No significant difference was observed between groups at each time point.
