# Supplementary material for: Effect of exercise training after bariatric surgery: A 5-year follow-up study of a randomized controlled trial
Source: PLoS One. 2022 Jul 15;17(7):e0271561. doi: 10.1371/journal.pone.0271561 (PMC9286216; doi:10.1371/journal.pone.0271561)
Supplement: S3 Table — Non-adjusted P-value from Wilcoxon rank sum test, χ2 test or Fisher exact test. (DOCX) [file pone.0271561.s003.docx]

**S3 Table. Comparisons between categories of weight regain between 1 year and 5 years after RYGB in terms of preoperative characteristics or RYGB-induced changes in body weight and body composition**

|  |  | **N** | **All patients**  **(N= 52)** |  | **Weight regain between 1 year and 5 years post-RYGB** | | |
| --- | --- | --- | --- | --- | --- | --- | --- |
|  |  |  |  |  | **<10 %**  **(N = 20)** | **≥10 %**  **(N= 32)** | **P-value** |
| **Intervention group**  ref: CON  PRO  PRO+EX |  | 52 | 16 (30.8%)  22 (42.3%)  14 (26.9%) |  | 5 (25.0%)  8 (40.0%)  7 (35.0%) | 11 (34.8%)  14 (43.8%)  7 (21.9%) | ref  0.74  0.30 |
| **Preoperative characteristics** |  |  |  |  |  |  |  |
| Age, y |  | 52 | 43.6 (10.1) |  | 45.2 (8.8) | 42.7 (10.8) | 0.37 |
| BMI, kg/m^2^ |  | 52 | 44.2 (5.5) |  | 43.4 (5.1) | 44.8 (5.7) | 0.36 |
| Body fat, % |  | 51 | 49.9 (3.8) |  | 50.6 (3.1) | 49.5 (4.2) | 0.27 |
| Type 2 diabetes, N (%) |  | 52 | 17 (32.7%) |  | 7 (35.0%) | 10 (31.3%) | 0.93 |
| Sleep apnea syndrome, N (%) |  | 52 | 29 (55.8%) |  | 9 (45.0%) | 20 (62.5%) | 0.38 |
| Hypertension, N (%) |  | 52 | 18 (34.6%) |  | 7 (35.0%) | 11 (34.4%) | 1 |
| **Outcomes 1 year after RYGB** |  |  |  |  |  |  |  |
| Weight loss, % |  | 52 | -32.3 (6.7) |  | -33.1 (5.3) | -31.7 (7.4) | 0.44 |
| Loss in fat mass, % |  | 51 | -46.5 (10.9) |  | -48.4 (10.6) | -45.4 (11.1) | 0.34 |
| Loss in lean body mass, % |  | 51 | -16.8 (5.9) |  | -14.9 (5.5) | -18.0 (5.9) | 0.07 |
| **Outcomes at last follow-up** |  |  |  |  |  |  |  |
| Time elapse since surgery, y |  | 52 | 5.7 (0.9) |  | 5.5 (0.8) | 5.8 (1.0) | 0.31 |
| Total weight loss, % |  | 52 | -27.7 (8.0) |  | -33.3 (4.6) | -24.2 (7.6) | **<0.001** |
| Loss in fat mass, % |  | 50 | -37.3 (12.9) |  | -47.1 (9.4) | -31.3 (11.1) | **<0.001** |
| Loss in lean body mass, % |  | 50 | -16.7 (6.1) |  | -17.7 (5.5) | -16.2 (5.8) | 0.39 |
| Type 2 diabetes, N (%) |  | 52 | 9 (17.3%) |  | 3 (15.0%) | 6 (18.9%) | 1 |
| Sleep apnea syndrome, N (%) |  | 52 | 5 (9.6%) |  | 1 (5.0%) | 4 (12.5%) | 0.64 |
| Hypertension, N (%) |  | 52 | 10 (19.2%) |  | 3 (15.0%) | 7 (21.9%) | 0.72 |

Non-adjusted P-value from Wilcoxon rank sum test, χ^2^ test or Fisher exact test.
