# Supplementary figures and images for: Effect of exercise training after bariatric surgery: A 5-year follow-up study of a randomized controlled trial
Source: PLoS One. 2022 Jul 15;17(7):e0271561. doi: 10.1371/journal.pone.0271561 (PMC9286216; doi:10.1371/journal.pone.0271561)

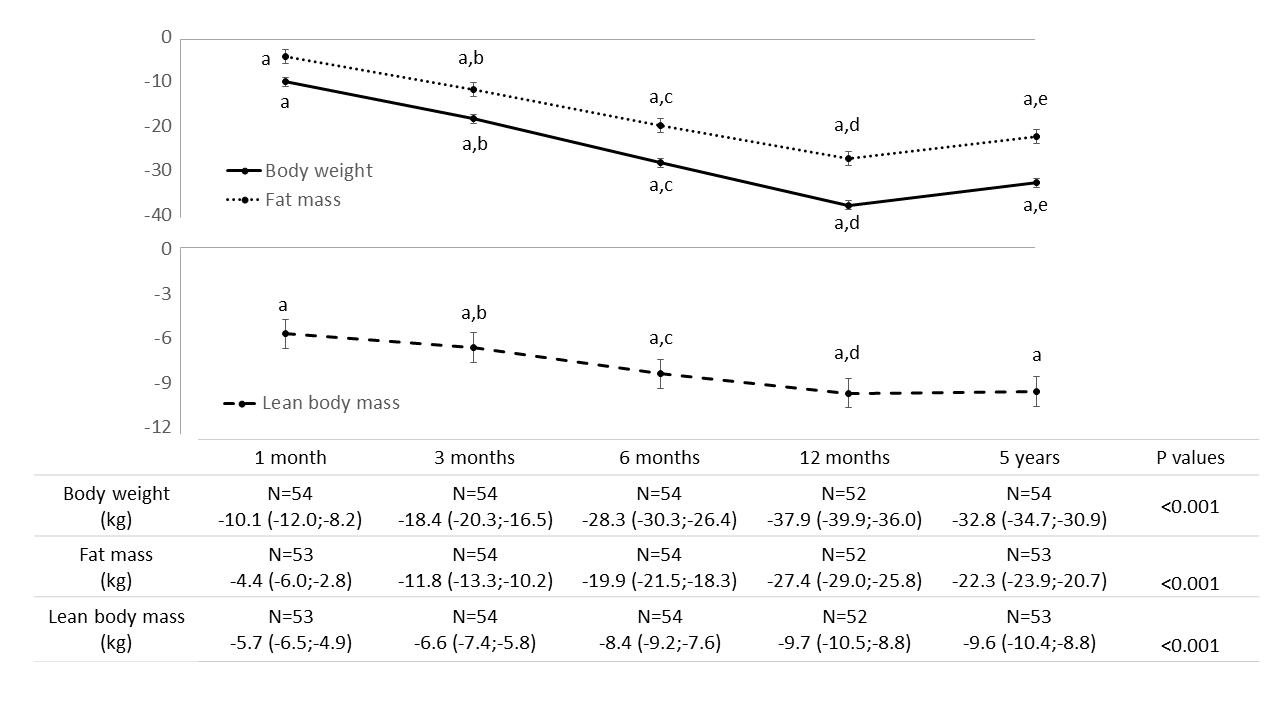

Supplement: S1 Fig — P values for time effect in mixed models. a Significantly different from preoperative value. b Significantly different from 1-month follow-up post-surgery. c Significantly different from 3-month follow-up post-surgery. d Significantly different from 6-month follow-up post-surgery. e Significantly different from 12-month follow-up post-surgery. (TIF) [file pone.0271561.s004.tif]
